# Vehicle Trajectory Reconstruction Method for Urban Arterial Roads Based on Multi-Source Data Fusion

**DOI:** 10.3390/s25072102

**Published:** 2025-03-27

**Authors:** Zhanhang Shi, Dong Guo, Lili Bian, Yvbin Liu, Bin Zhou, Feng Sun

**Affiliations:** 1 School of Transportation and Vehicle Engineering, Shandong University of Technology, Zibo 255000, China; 22402030005@stumail.sdut.edu.cn (Z.S.); guodong@sdut.edu.cn (D.G.); 24402030182@stumail.sdut.edu.cn (Y.L.); 2 Zibo Transportation Construction and Development Center, Zibo 255000, China; bianlili2000@163.com; 3 State Key Lab of Intelligent Transportation System, Beijing 100088, China; binzhou@buaa.edu.cn

**Keywords:** signal-controlled roads, vehicle trajectory reconstruction, fixed sensors, probe vehicles, car-following model, particle filtering

## Abstract

Vehicle trajectory data contain extensive spatiotemporal information and are of great significance for analyzing the operational patterns of urban traffic networks, optimizing traffic signal control and achieving refined traffic management. However, due to the low penetration rate of probe vehicles and the limited coverage of fixed sensors, it remains challenging to obtain comprehensive full-sample vehicle trajectory data. To address this issue, this paper proposes a multi-source data fusion-based vehicle trajectory reconstruction method, which comprises vehicle trajectory state estimation and a self-optimization algorithm. First, the trajectory states of undetected vehicles are categorized into four types based on the trajectory states of adjacent probe vehicles. Four corresponding trajectory estimation models are then established using an extended Intelligent Driver Model to reconstruct the initial trajectories of undetected vehicles. Second, a particle filter-based trajectory self-optimization algorithm is proposed, integrating upstream and downstream fixed sensor data to iteratively correct and optimize the initial trajectories by minimizing errors, thereby enhancing trajectory accuracy and smoothness. Case studies demonstrate that the proposed method achieves outstanding performance under low PV penetration rates and in complex traffic environments. Compared to baseline methods, MAE, MAPE, and RMSE are reduced by 14.31%, 22.87%, and 13.36%, respectively. Furthermore, the reconstruction errors of the proposed method gradually decrease as traffic density and PV penetration rates increase. Notably, PV penetration has a more significant impact on model accuracy. These findings confirm the robustness and effectiveness of the proposed method in complex traffic scenarios, providing critical technical support for refined urban traffic management and optimized decision-making.

## 1. Introduction

Vehicle trajectory data, as a crucial source of traffic information, encapsulates rich spatiotemporal characteristics [1] and has been widely applied in fields such as traffic state estimation, traffic flow modeling, signal control optimization, and the evaluation of traffic energy consumption and emissions [2,3]. However, in real-world scenarios, directly obtaining complete vehicle trajectory samples through methods like video cameras and drones is a resource-intensive and time-consuming task [4]. Moreover, existing fixed and mobile sensors, due to their limited deployment and penetration rates, can only provide partial trajectory data, which is insufficient for accurately describing complex urban traffic flow [5]. Therefore, the accurate reconstruction of full vehicle trajectories on urban arterial roads using multi-source detection data has become an increasingly important research challenge.

Most existing trajectory reconstruction methods rely on traffic flow information collected by sensors, which are primarily classified into two types: fixed sensors and mobile sensors. Fixed sensors (such as inductive loops and video surveillance) provide discrete data at specific locations, such as traffic flow [6], queue length [7], and travel time [8]. These sensors have been widely deployed on urban arterials and highways, providing valuable data for numerous studies [9,10]. However, due to the limited detection range of fixed sensors, which is constrained by their installation locations and their relatively low deployment density, they cannot comprehensively capture the spatiotemporal information of vehicles. This leads to a low spatiotemporal resolution and the inability to fully reflect the dynamic changes in traffic flow. Consequently, data derived from fixed sensors cannot offer a complete representation of the operational state of the entire traffic system, especially in complex urban traffic environments.

In contrast, mobile sensors (such as probe vehicles (PVs) and connected vehicles (CVs)) are capable of providing high-resolution, spatiotemporally continuous data, which has led to their widespread use in various studies, including traffic flow estimation [11], the development of basic graph models [12], and traffic signal control optimization [13]. By tracking the precise trajectories of vehicles, mobile sensors can capture more detailed traffic flow information, enabling a more accurate reconstruction of dynamic traffic flow variations [14]. However, the low penetration rate of mobile sensors means that the trajectory data they collect represent only a small portion of the overall traffic flow. Therefore, researchers often rely on simplified traffic flow models (such as variational theory (VT)) to reconstruct complete vehicle trajectories [15,16], compensating for the limitations in data collection and providing support for a comprehensive understanding of the entire traffic system’s operational state.

Moreover, with the rapid development of intelligent transportation systems and vehicular network technologies, connected and autonomous vehicles (CAVs) have demonstrated significant potential in the fields of traffic data collection and trajectory reconstruction. By integrating various sensors and vehicular communication technologies, CAVs can obtain real-time, high-precision vehicle location information and dynamic behavior data [17]. Existing studies have shown that high-penetration vehicle trajectory samples have been successfully used for estimating macroscopic traffic states, such as periodic flow and queue length, with notable success [18]. However, due to the currently low penetration rate of CAVs and the complexity of driving behaviors, the data collected remain limited, making it difficult to obtain fully sampled vehicle trajectory data.

The above studies clearly reveal several challenges faced in accurately reconstructing vehicle trajectories using data from fixed and mobile sensors, which can be summarized as follows:

(1) Fixed sensors can only capture local traffic state information at their deployment locations, making it difficult to fully reflect the dynamic traffic flow characteristics of the entire urban road network. In particular, the current deployment density of fixed sensors is relatively low and primarily concentrated near intersections, resulting in significant spatial coverage gaps along urban road segments.

(2) In real urban road environments, the penetration rate of probe vehicles (PVs) remains relatively low due to high equipment costs and deployment challenges, resulting in insufficient trajectory data coverage. Moreover, existing studies often rely on modeling based on uniformly distributed vehicle trajectories, failing to accurately capture the stochastic distribution characteristics of vehicle trajectories under complex traffic conditions, which significantly limits the accuracy and applicability of trajectory reconstruction.

(3) Fixed sensors typically collect cross-sectional traffic flow data, which reflect the traffic state at specific locations, whereas PVs provide continuous trajectory data with both spatial and temporal dimensions. The differences in data structure and spatiotemporal resolution between these two data sources introduce significant irregularities in the trajectory reconstruction process.

Based on the aforementioned challenges, this paper proposes a multi-source data fusion-based vehicle trajectory reconstruction method to enhance the accuracy and practicality of vehicle trajectory reconstruction on urban arterial roads. This method effectively integrates the advantages of data from fixed sensors and probe vehicles (PVs), overcoming the limitations of single data sources in terms of spatial coverage and trajectory continuity. It not only resolves the spatial coverage gaps caused by the limited deployment of fixed sensors and the insufficient data coverage resulting from low PV penetration rates but also addresses the data format incompatibility between different data sources. This approach is particularly suitable for complex traffic environments characterized by sparse fixed sensor deployment and low PV penetration rates.

Through deep multi-source data fusion and model optimization, the proposed method can reconstruct more complete and accurate vehicle trajectories. In practical applications, the reconstructed trajectory data provide strong support for traffic management, which is reflected in the following three aspects: First, the system can dynamically optimize traffic signal timing strategies based on real-time trajectory data, effectively alleviating traffic congestion and improving traffic efficiency. Second, by monitoring and analyzing trajectory data in real time, the system can quickly identify and respond to traffic anomalies (such as traffic accidents or vehicle breakdowns), significantly reducing emergency response times. Finally, the reconstructed trajectory data provide accurate support for traffic flow trend analyses and travel pattern studies, facilitating more refined and intelligent traffic management.

The remainder of this paper is organized as follows: Section 2 provides a brief review of the related literature on vehicle trajectory reconstruction in different data source environments. Section 3 describes the research problem. Section 4 presents the overall framework and the main trajectory reconstruction algorithms. Section 5 validates the applicability of the proposed method under different traffic densities and PV penetration rates through actual survey data and simulation experiments. Finally, Section 6 concludes this paper and discusses potential directions for future research.

## 2. Literature Review

In recent years, vehicle trajectory reconstruction has garnered significant attention. To enable trajectory reconstruction across different data source environments, various methods have been proposed. These methods have been applied to scenarios including both continuous and discontinuous traffic flow facilities, such as highways and urban intersections. In this section, existing trajectory reconstruction studies are classified based on the data sources used.

### 2.1. Fixed Sensor Data

Fixed sensors have been widely applied in queue length estimation [7,19], travel time estimation [20], and path flow reconstruction [21]. However, studies focused solely on using fixed sensor data for precise vehicle trajectory reconstruction are relatively scarce. According to existing research, shockwave theory is commonly employed, where traffic flow is decomposed into the arrival and departure of individual vehicles to estimate their trajectories [22]. Given that fixed sensors can only provide detection at fixed locations, some complex vehicle behaviors, such as car-following, acceleration, and deceleration, are often neglected. Although such single-point detection data have shown good performance in travel time estimation, reconstructed trajectories are not suitable for more microscopic applications, such as fuel consumption and traffic flow fluctuations. Additionally, the deployment location and data quality of fixed sensors have a significant impact on the applicability of methods. Consequently, more researchers have begun to focus on addressing the issues of missed and over-detected events in fixed sensor data to improve the robustness of their methods [23].

### 2.2. Mobile Sensor Data

With the rapid development of PVs and connected vehicle technologies, methods based on mobile sensors have gained increasing attention. Although these methods overcome the limitations of fixed sensors in terms of the detection range, they face challenges such as a low upload frequency and penetration rate. To address the issue of low upload frequencies in PVs, Wei et al. [8] proposed a method based on particle filtering (PF) for the high-resolution trajectory reconstruction of individual vehicles. The driving behaviors were categorized into four types, acceleration, deceleration, cruising, and idling, and the optimal sequence was selected based on probability theory. To address the low penetration rate of PVs, traffic flow models, such as variational theory (VT) and car-following models (CFs), have been used to reconstruct fully sampled trajectories. However, VT neglects the random fluctuations in speed, which leads to reconstructed trajectories exhibiting constant acceleration characteristics [16]. While the CF model can approximately capture acceleration and deceleration behaviors, a higher penetration rate is required to reduce the accumulation of estimation errors [24].

Although existing studies have proposed incorporating public transport and taxis as additional PVs to increase PV penetration rates, differences in driving patterns between different types of vehicles may lead to inconsistencies in behavioral and spatiotemporal characteristics. Moreover, public transport follows fixed routes, which may not accurately reflect real-time traffic conditions, thereby affecting the accuracy of trajectory reconstruction. Therefore, relying solely on mobile sensor data for vehicle trajectory reconstruction still presents significant challenges.

### 2.3. Connected and Automated Vehicle Data

Due to the limitations of fixed and mobile sensors, some researchers have turned to vehicle trajectory reconstruction in the environment of connected and autonomous vehicles (CAVs). For instance, Wang et al. [25] used the Wiedemann model to estimate the driving behavior of vehicles within the detection range of CAVs and combined it with cellular automata to reconstruct the complete trajectories of mixed traffic flow consisting of both CAVs and manually driven vehicles. To better capture the microscopic driving behaviors of vehicles, Chen et al. (2022) [26] applied an improved Intelligent Driver Model (IDM) to estimate the positions and speeds of undetected vehicles between consecutive CAVs in mixed traffic flow, thereby reconstructing the full trajectories of these undetected vehicles. In the context of signal-controlled intersections, a complex traffic environment, Chen et al. (2022) [27] proposed an extended car-following model to reconstruct the trajectories of undetected vehicles in such settings. However, these vehicles are typically used in specific scenarios, and their penetration rate in real urban roads remains low. As a result, they can only collect specific trajectory data within a limited time frame in vehicle data acquisition environments.

### 2.4. Multi-Source Data

To overcome the limitations of single data sources, multi-source data fusion has garnered widespread attention in recent years. Compared to traditional vehicle trajectory reconstruction methods based on a single data source, multi-source data fusion methods offer higher accuracy in vehicle trajectory reconstruction [28]. This is particularly true in applications such as energy consumption analysis and traffic state estimation, where the fusion of heterogeneous data has yielded promising results [29,30]. Existing research has utilized the fusion of fixed sensor data with mobile sensor data to estimate vehicle movement states on highways [31]. By integrating data from different sensors (e.g., GPSs and cameras), it is possible to comprehensively perceive traffic conditions, enhancing prediction accuracy and real-time responsiveness. Fusion techniques, such as Kalman filtering and particle filtering, have demonstrated excellent performance in handling data missingness, noise interference, and sensor errors [32]. However, these methods primarily focus on estimating travel time and are often associated with high computational complexity and lower accuracy. Therefore, the fusion of heterogeneous data sources still faces significant challenges.

In summary, existing vehicle trajectory reconstruction methods have not fully leveraged multi-source data. Fixed sensor data are typically used as a constraint for travel time estimation, while data recorded by PVs suffer from error accumulation due to the limitations of the CF model. Therefore, in environments characterized by spatiotemporally sparse data from both fixed and mobile sensors, accurately reconstructing full-sample vehicle trajectories remains a significant challenge.

## 3. Problem Description

**Figure 1:**
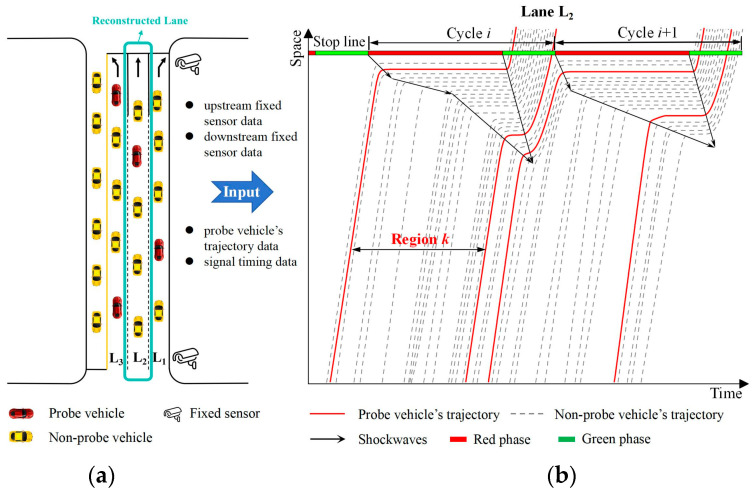
Data environment and reconstruction targets in the study area: (**a**) trajectory reconstruction scope; (**b**) trajectory area segmentation.

The multi-source data environment studied in this paper includes three types of data: fixed detection data, mobile monitoring data, and signal timing data. As shown in Figure 1a, fixed sensors are installed at upstream and downstream locations of urban road segments and continuously record the arrival time and instantaneous speed of each vehicle on lane L_2_. Additionally, PVs are randomly distributed on the road, but their penetration rate is relatively low, as illustrated by the red lines in Figure 1b. Considering that fixed sensors on urban roads are typically deployed at the entrance and exit lanes of intersections, the reconstruction scope in this study is defined as the monitored blind-zone road segments between adjacent intersections on urban roads.

In Figure 1b, the gray dashed lines represent the trajectories of undetected vehicles without mobile sensors, which are the reconstruction targets of this study. However, due to the placement of fixed sensors at the upstream and downstream ends of road segments, full coverage of the segment cannot be achieved. Additionally, the number of probe vehicle trajectories is significantly lower than that of undetected vehicles, making it challenging to reconstruct full-sample vehicle trajectories in such a sparse detection data environment. Furthermore, these sensors are incapable of capturing the precise locations and processes of lane changes or overtaking maneuvers. Therefore, this study assumes that all undetected vehicles follow a car-following behavior with the preceding vehicle.

To simplify the reconstruction process, this study divides the spatiotemporal map into multiple regions based on the trajectories of PVs and reconstructs the trajectories sequentially for each region. Each region corresponds to the spatial range between two consecutive PVs, as shown in Figure 1b. In the following Section 4, the focus will be on how to reconstruct trajectories within a single divided region, with similar approaches applicable to other regions. It is important to note that no significant differences exist between the data collected by fixed sensors and that collected by PVs in this study, ensuring the avoidance of missed detections and duplicate counts.

## 4. Methods

### 4.1. General Framework

**Figure 2:**
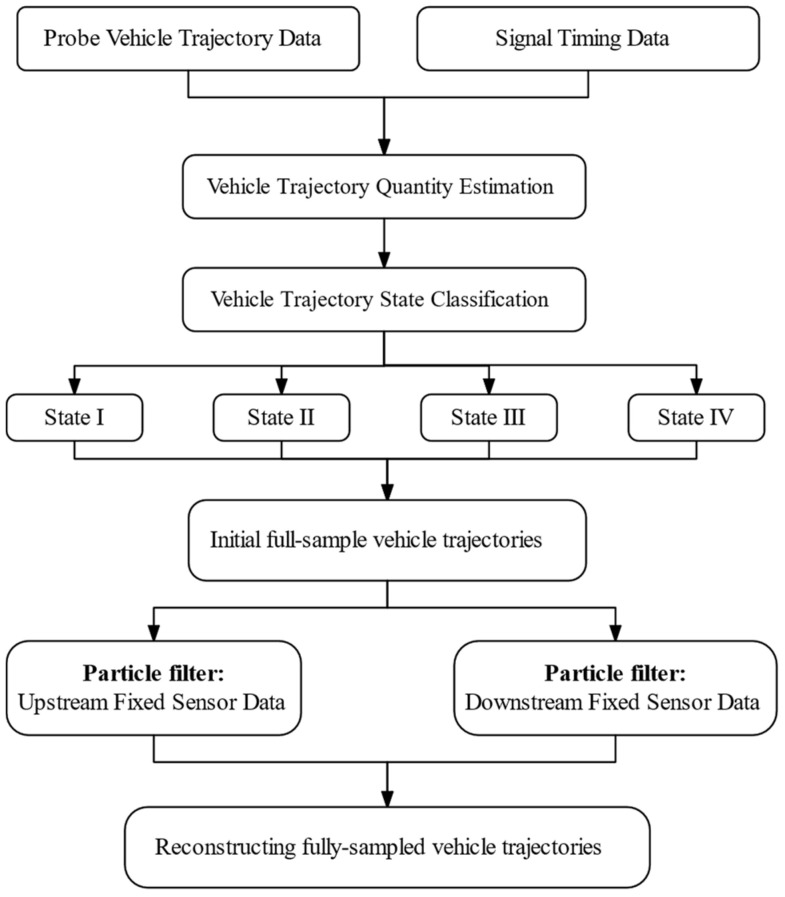
Flowchart of the proposed trajectory reconstruction method.

To accurately reconstruct the full-sample trajectories of all vehicles in environments characterized by low deployment rates of fixed sensors and low penetration rates of PVs, this paper proposes a trajectory reconstruction algorithm framework. The framework consists of two main components: a vehicle trajectory state estimation algorithm and a trajectory self-optimization algorithm. The initial full-sample trajectories of undetected vehicles are estimated using an extended car-following model based on the trajectory data of adjacent PVs. Then, an improved particle filter algorithm is employed to integrate and optimize the initial trajectories with fixed detection data, resulting in accurate full-sample vehicle trajectories. Figure 2 illustrates the overall flowchart of the proposed method.

In the vehicle trajectory state estimation algorithm, the spatiotemporal characteristics of adjacent PVs are analyzed to classify trajectories into four distinct states. Corresponding trajectory estimation models are then applied to each state to reconstruct the trajectories, resulting in the initial full-sample vehicle trajectories (see Section 4.2 for details).

In the vehicle trajectory correction and optimization algorithm, a particle filtering approach is employed to fully integrate detection data from upstream and downstream fixed sensors. This process refines and optimizes the initial full-sample vehicle trajectories, addressing the limitations of PVs in terms of coverage and randomness. The aim is to enhance the accuracy of trajectory reconstruction (see Section 4.3 for details).

### 4.2. Vehicle Trajectory State Estimation Algorithm

As illustrated in Figure 1b, a significant number of undetected vehicles are present between consecutive PVs. Therefore, it is necessary to first estimate the number of undetected vehicles based on the trajectories of PVs. Let the leading vehicle in each reconstruction region be denoted as $V_{i-1}^{\mathrm{pv}}$ and the following vehicle as $V_{i}^{\mathrm{pv}}$. Considering the minimum safe spacing between vehicles, the maximum possible number of undetected vehicles *m* between adjacent PVs is represented as M, as shown in Equation (1) [33]

(1) $$M=\mathrm{ent}\left(\underset{t\in [0,\Delta t,2\Delta t,\ldots ,k\Delta t]}{\min}\frac{x_{i-1}^{\mathrm{pv}}(t)-x_{i}^{\mathrm{pv}}(t)}{s_{0}+l}\right)$$

where $x_{i-1}^{\mathrm{pv}}(t)$ represents the position of vehicle $V_{i-1}^{\mathrm{pv}}$ at time *t*, and $x_{i}^{\mathrm{pv}}(t)$ represents the position of vehicle $V_{i}^{\mathrm{pv}}$ at time *t*. Here, *l* denotes the average vehicle length, $s_{0}$ is the minimum standstill spacing, Δ*t* represents the unit time step, and *k* is the total number of time steps.

The undetected vehicles between $V_{i-1}^{\mathrm{pv}}$ and $V_{i}^{\mathrm{pv}}$ are denoted as $V_{i-1,j}^{\mathrm{npv}}$, where *j* = 1, 2,…, *m*. Among these, $V_{i-1,m}^{\mathrm{npv}}$ serves as the leading vehicle for $V_{i}^{\mathrm{pv}}$, and the two vehicles exhibit car-following behavior. Based on the car-following model, the reconstructed acceleration ${\hat{a}}_{i}^{\mathrm{pv}}(t)$ of vehicle $V_{i}^{\mathrm{pv}}$ can be derived from the trajectory data of $V_{i-1,m}^{\mathrm{npv}}$. The calculation formula is provided in Equation (2).

(2) $${\hat{a}}_{i}^{\mathrm{pv}}(t)=a_{\max}\left[1-{\left(\frac{v_{i}^{\mathrm{pv}}(t)}{v_{0}}\right)}^{4}-{\left(\frac{s_{0}+v_{i}^{\mathrm{pv}}(t)T+\frac{v_{i}^{\mathrm{pv}}(t)[v_{i}^{\mathrm{pv}}(t)-v_{i-1,m}^{\mathrm{npv}}(t)]}{2\sqrt{a_{\max}b}}}{x_{i-1,m}^{\mathrm{npv}}(t)-x_{i}^{\mathrm{pv}}(t)}\right)}^{2}\right]$$

where $v_{i}^{\mathrm{pv}}\left(t\right)$ represents the speed of vehicle $V_{i}^{\mathrm{pv}}$ at time *t*, and $v_{i-1,m}^{\mathrm{npv}}(t)$ represents the speed of vehicle $V_{i-1,m}^{\mathrm{npv}}$ at the same time. Similarly, $x_{i-1,m}^{\mathrm{npv}}(t)$ represents the position of vehicle $V_{i-1,m}^{\mathrm{npv}}$ at time *t*. $v_{0}$ represents the desired speed, $a_{\max}$ denotes the maximum acceleration, *T* corresponds to the safe time headway, *b* indicates the absolute value of the comfortable deceleration, and $s_{0}$ signifies the minimum standstill spacing.

Here, the root-mean-square error (RMSE) between the reconstructed acceleration and the actual acceleration of $V_{i}^{\mathrm{pv}}$ is used as the evaluation criterion. At time *t*, the number of undetected vehicles m(t) that minimizes the error is considered the optimal vehicle count for that moment. By aggregating the optimal vehicle count across all time steps, the most frequently occurring optimal vehicle count is identified as the final optimal number of undetected vehicles m∗ between adjacent PVs. The calculation formula is provided in Equation (3).

(3) $$m*=\min\left\{M,\mathrm{MODE}\left(\arg\underset{m}{\min}\sqrt{\frac{1}{n}\sum_{i=1}^{n}{\left({\hat{a}}_{i}^{\mathrm{pv}}(t)-a_{i}^{\mathrm{pv}}(t)\right)}^{2}}\right)\right\},\forall t\in \left\{t_{1},t_{2},\ldots ,t_{k}\right\}$$

**Figure 3:**
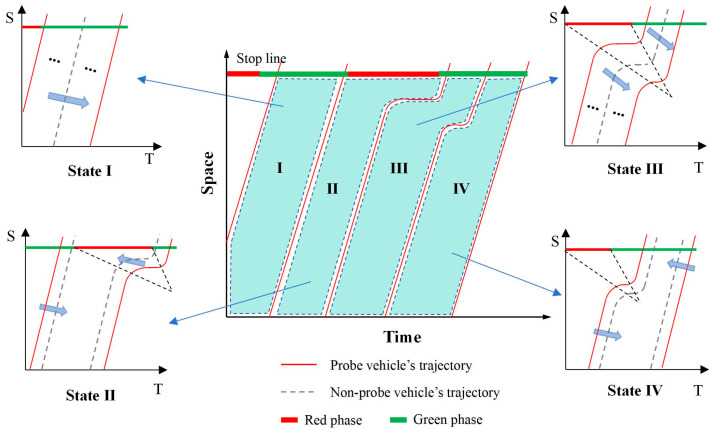
State-based vehicle trajectory reconstruction.

After determining the optimal number of undetected vehicles, the trajectories of undetected vehicles are classified into the following four categories based on the trajectory states of adjacent PVs: ① both the leading and following vehicles do not experience queuing, as shown in “State I” in Figure 3; ② the leading vehicle does not experience queuing, while the following vehicle does, as shown in “State II” in Figure 3; ③ both the leading and following vehicles experience queuing, as shown in “State III” in Figure 3; and ④ the leading vehicle experiences queuing, while the following vehicle does not, as shown in “State IV” in Figure 3.

All undetected vehicle trajectories may involve these four types; however, traditional car-following models can only estimate the trajectories of undetected vehicles in “State I” and “State III”. For the other two types, it is necessary to extend the car-following model into the spatiotemporal dimension to estimate the leading vehicle’s trajectory based on the following vehicle, thereby reconstructing the trajectories of undetected vehicles. This study adopts the Intelligent Driver Model (IDM) as the base car-following model, as it has been extensively validated and is capable of simulating traffic flow patterns with high fidelity under various traffic conditions. Notably, IDM demonstrates high accuracy in car-following modeling, even in scenarios with low mobile sensor penetration rates [27].

(1) State I: both the leading and following vehicles do not experience queuing.

When both the leading vehicle $V_{i-1}^{\mathrm{pv}}$ and the following vehicle $V_{i}^{\mathrm{pv}}$ have not experienced queuing upon arrival, the reconstructed trajectories of undetected vehicles are assumed to fully adhere to the car-following model. After determining the optimal number of undetected vehicles m∗, the IDM model can be directly applied to smoothly estimate the trajectories of undetected vehicles. Taking the undetected vehicle $V_{i-1,m}^{\mathrm{npv}}$ as an example, changes in the trajectory of $V_{i-1,m}^{\mathrm{npv}}$ will affect the trajectory of the following vehicle $V_{i}^{\mathrm{pv}}$. Assuming the trajectory of the adjacent vehicle $V_{i}^{\mathrm{pv}}$ at time *t* is known, the trajectory of vehicle $V_{i-1,m}^{\mathrm{npv}}$ at time *t* can be formulated as an optimization problem, as shown in Equation (4).

(4) $$\begin{array}{l} \min f(x_{i-1,m}^{\mathrm{npv}}(t))=\sqrt{\frac{1}{k}\sum_{t=0}^{k\Delta t}{\left[{\hat{a}}_{i}^{\mathrm{pv}}(t)-a_{i}^{\mathrm{pv}}(t)\right]}^{2}}\\ s.t.\;v_{i-1,m}^{\mathrm{npv}}(t)=v_{i}^{\mathrm{pv}}(t)-\left[v_{i-1}^{\mathrm{pv}}(t)-v_{i}^{\mathrm{pv}}(t)\right]{\left(m*+1\right)}^{-1}\leq v_{0}\\ \;\;\;\;\;\;\;x_{i-1,m}^{\mathrm{npv}}(t)=x_{i}^{\mathrm{pv}}(t)-\left[x_{i-1}^{\mathrm{pv}}(t)-x_{i}^{\mathrm{pv}}(t)\right]{\left(m*+1\right)}^{-1}\\ \;\;\;\;\;\;\;x_{i}^{\mathrm{pv}}(t)+s_{0}+l\leq x_{i-1,m}^{\mathrm{npv}}(t)\leq x_{i-1}^{\mathrm{pv}}(t)-s_{0}-l\\ \;\;\;\;\;\;\;{\hat{a}}_{i}^{\mathrm{pv}}(t)=f\left(v_{i}^{\mathrm{pv}}\left(t\right),v_{i-1,m}^{\mathrm{npv}}\left(t\right)-v_{i}^{\mathrm{pv}}\left(t\right),x_{i-1,m}^{\mathrm{npv}}\left(t\right)-x_{i}^{\mathrm{pv}}\left(t\right)\right) \end{array}$$

where $a_{i}^{\mathrm{pv}}(t)$ represents the actual acceleration of vehicle $V_{i}^{\mathrm{pv}}$ at time *t*.

The objective function is defined to minimize the root-mean-square error (RMSE) between the reconstructed acceleration ${\hat{a}}_{i}^{\mathrm{pv}}(t)$ and the actual acceleration $a_{i}^{\mathrm{pv}}(t)$, which is used to determine the position of $x_{i-1,m}^{\mathrm{npv}}(t)$. Additionally, the speed of vehicle $V_{i-1,m}^{\mathrm{npv}}$ at time t can also be obtained using this algorithm.

Similarly, the trajectories of vehicle $V_{i-1,m}^{\mathrm{npv}}$ at time points *t* + Δ*t*, *t* + 2Δ*t*, …, and *t* + *k*Δ*t* can all be obtained using this algorithm. The Intelligent Driver Model (IDM), due to its simple equations and clear logic, is well-suited for spatiotemporal extension. Consequently, it can effectively reconstruct the trajectories of undetected vehicles under “State II” and “State IV”.

(2) State II: the leading vehicle does not experience queuing, while the following vehicle does.

When the leading vehicle $V_{i-1}^{\mathrm{pv}}$ does not experience queuing upon arrival, but the following vehicle $V_{i}^{\mathrm{pv}}$ does, the trajectories to be reconstructed are categorized into two types: vehicles that have not experienced queuing and vehicles that have. Based on the stopping position of the following vehicle, the number of queued vehicles ahead can be calculated, and the total number of undetected vehicles to be reconstructed can be determined using Equation (3). Consequently, the numbers of queued and non-queued vehicles in the reconstructed trajectories can be calculated using the following equations:

(5) $$m_{\mathrm{stop}}=\frac{\left|x_{\mathrm{stopline}}-x_{i}^{\mathrm{stop}}\right|}{s_{0}+l}$$

where $m_{\mathrm{stop}}$ represents the number of vehicles that have experienced stopping, $x_{\mathrm{stopline}}$ denotes the position of the stop line at the intersection, and $x_{i}^{\mathrm{stop}}$ indicates the stopping position of the following vehicle.

The number of vehicles that have not experienced stopping is given by Equation (6).

(6) $$m_{\mathrm{no}-\mathrm{stop}}=m^{*}-m_{\mathrm{stop}}$$

where $m_{\mathrm{no}-\mathrm{stop}}$ represents the number of vehicles that have not experienced stopping.

**Figure 4:**
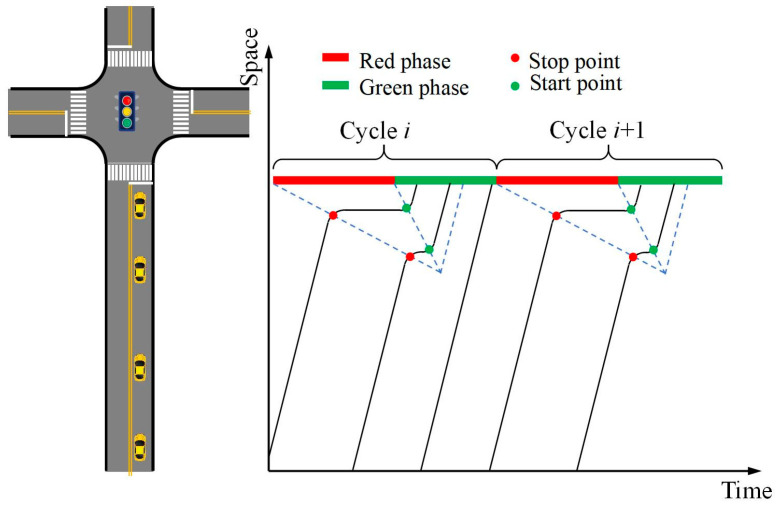
Schematic diagram of start–stop points.

The trajectories of vehicles experiencing the queuing process can be divided into three phases: following the leading vehicle, queuing while waiting, and departing after the leading vehicle. Among these, the trajectory during the phase of following the leading vehicle exhibits dynamic characteristics similar to the trajectories of vehicles that do not experience queuing, as well as those in “State I”, as illustrated in Figure 4. Consequently, these two types of trajectories can be reconstructed using Equation (4). The process for solving the trajectories during the remaining phases is as follows:

a. Reconstructing trajectories during the queuing phase.

When vehicles queue at an intersection, stop waves and start waves are generated. Based on traffic wave theory, the queuing trajectories of vehicles can be calculated, as illustrated in Figure 4. In the queuing phase, the leading vehicle is denoted as $V_{i-1}^{\mathrm{pv}}$ and the following vehicle as $V_{i}^{\mathrm{pv}}$. Taking $V_{i-1,m}^{\mathrm{npv}}$ as an example, the positions and times of its stopping and starting points can be computed as follows:

(7) $$x_{i-1,m}^{\mathrm{npv}-p}=x_{i-1,m}^{\mathrm{npv}-s}=x_{i-1}^{\mathrm{pv}-p}-s_{0}-l$$

(8) $$t_{i-1,m}^{\mathrm{npv}-p}=\frac{x_{i-1}^{\mathrm{pv}-p}-s_{0}-l-b^{p}}{w^{p}}$$

(9) $$t_{i-1,m}^{\mathrm{npv}-s}=\frac{x_{i-1}^{\mathrm{pv}-s}-s_{0}-l-b^{s}}{w^{s}}$$

where $x_{i-1,m}^{\mathrm{npv}-p}$ and $t_{i-1,m}^{\mathrm{npv}-p}$ represent the stopping position and time of vehicle $V_{i-1,m}^{\mathrm{npv}}$, respectively; $x_{i-1,m}^{\mathrm{npv}-s}$ and $t_{i-1,m}^{\mathrm{npv}-s}$ represent the starting position and time of vehicle $V_{i-1,m}^{\mathrm{npv}}$, respectively; $w^{p}$ represents the speed of the stopping wave, $w^{s}$ represents the speed of the dissipating wave, $b^{p}$ is the intercept of the stopping wave line, and $b^{s}$ is the intercept of the starting wave line.

b. Reconstructing trajectories during the following and departure phase.

For the trajectory reconstruction during the phase of following and departing after the leading vehicle, the CF model cannot be directly applied due to the unavailability of the leading vehicle’s trajectory. However, since the trajectory of the following vehicle is known, an extended car-following model can be employed to estimate the missing trajectory of the leading vehicle. Under high-density traffic flow conditions, there is a strong correlation between the trajectories of the leading and following vehicles. When the trajectory of vehicle $V_{i-1,m}^{\mathrm{npv}}$ at time *t* is known, its trajectory at time *t* + Δ*t* can be calculated using the following equation [27]:

(10) $$\begin{array}{l} \min location\;error=\sqrt{{\left({\hat{x}}_{i}^{\mathrm{pv}}(t+2\Delta t)-x_{i}^{\mathrm{pv}}(t+2\Delta t)\right)}^{2}}\\ s.t.\;{\hat{a}}_{i}^{\mathrm{pv}}(t+\Delta t)=a_{\max}\left[1-{\left(\frac{v_{i}^{\mathrm{pv}}(t+\Delta t)}{v_{0}}\right)}^{4}-{\left(\frac{s^{*}(v_{i}^{\mathrm{pv}}(t+\Delta t),{\hat{v}}_{i-1,m}^{\mathrm{npv}}(t+\Delta t))}{{\hat{x}}_{i-1,m}^{\mathrm{npv}}(t+\Delta t)-x_{i}^{\mathrm{pv}}(t+\Delta t)}\right)}^{2}\right]\\ \max(v_{i-1,m}^{\mathrm{npv}}(t)-a_{\max}\Delta t,0)\leq {\hat{v}}_{i-1,m}^{\mathrm{npv}}(t+\Delta t)\leq \min(v_{i-1,m}^{\mathrm{npv}}(t)+a_{\max}\Delta t,v_{0})\\ {\hat{x}}_{i-1,m}^{\mathrm{npv}}(t+\Delta t)=x_{i-1,m}^{\mathrm{npv}}(t)+\frac{1}{2}({\hat{v}}_{i-1,m}^{\mathrm{npv}}(t+\Delta t)+v_{i-1,m}^{\mathrm{npv}}(t))\Delta t\\ {\hat{x}}_{i}^{\mathrm{pv}}(t+2\Delta t)=x_{i}^{\mathrm{pv}}(t+\Delta t)+v_{i}^{\mathrm{pv}}(t+\Delta t)\Delta t+\frac{1}{2}{\hat{a}}_{i}^{\mathrm{pv}}(t+\Delta t)\Delta t^{2} \end{array}$$

where $\;{\hat{x}}_{i-1,m}^{\mathrm{npv}}(t+\Delta t)$ represents the reconstructed position of vehicle $V_{i-1,m}^{\mathrm{npv}}$ when its speed is ${\hat{v}}_{i-1,m}^{\mathrm{npv}}(t+\Delta t)$, while ${\hat{x}}_{i}^{\mathrm{pv}}(t+2\Delta t)$ and ${\hat{a}}_{i}^{\mathrm{pv}}(t+\Delta t)$ denote the reconstructed position and reconstructed acceleration estimated using the IDM under the speed ${\hat{v}}_{i-1,m}^{\mathrm{npv}}(t+\Delta t)$. Additionally, ${\hat{v}}_{i-1,m}^{\mathrm{npv}}(t+\Delta t)$ indicates the actual position of vehicle $V_{i}^{\mathrm{pv}}$ at time *t* + 2Δ*t*, and $s^{*}$ represents the desired minimum spacing with the leading vehicle.

The objective function is defined as minimizing the squared error between the reconstructed position and the actual position. The algorithm requires the distance between two vehicles to remain small while ensuring compliance with the minimum safe distance. Similarly, the missing trajectories of other queued vehicles can also be reconstructed and completed using this algorithm, provided the above conditions are satisfied.

(3) State III: both the leading and following vehicles experience queuing.

Unlike traffic flows on highways, urban traffic flows are characterized by queuing phases, where the stopping positions of vehicles hold significant value for trajectory correction. Based on known trajectory information, the stopping positions of the leading and following queued vehicles are analyzed to adjust the number of intermediate vehicles inserted when both the leading and following vehicles are at a complete stop. The stopping position of vehicle $V_{i}^{\mathrm{pv}}$ is denoted as $x_{i}^{\mathrm{pv}}$, and that of vehicle $V_{i-1}^{\mathrm{pv}}$ is denoted as $x_{i-1}^{\mathrm{pv}}$. Using Equation (8), the corrected $m^{*}$ is applied to determine the number of undetected vehicles (m) that require reconstruction.

(11) $$m_{\mathrm{III}}=\frac{\left|x_{i}^{\mathrm{pv}}-x_{i-1}^{\mathrm{pv}}\right|}{s_{0}+l}$$

(12) $$m=\min\left\{m*,m_{\mathrm{III}}\right\}$$

When both the leading and following vehicles have experienced queuing upon arrival, the trajectories of the vehicles requiring reconstruction will also involve three phases during their movement: following the leading vehicle, queuing while waiting, and departing after the leading vehicle. The trajectories during the first phase, following the leading vehicle, can be reconstructed using the method for “State I”. For the second phase, queuing while waiting, the trajectories can be reconstructed using Equations (7)–(9). Finally, the trajectories during the third phase can be directly obtained using the car-following model, with the calculation formula provided as follows:

(13) $$a_{i-1,m}^{\mathrm{npv}}(t+\Delta t)=a_{\max}\left[1-{\left(\frac{v_{i-1,m}^{\mathrm{npv}}(t+\Delta t)}{v_{0}}\right)}^{4}-{\left(\frac{s^{*}({\hat{v}}_{i-1,m}^{\mathrm{npv}}(t+\Delta t),v_{i}^{\mathrm{pv}}(t+\Delta t))}{{\hat{x}}_{i}^{\mathrm{pv}}(t+\Delta t)-x_{i-1,m}^{\mathrm{npv}}(t+\Delta t)}\right)}^{2}\right]$$

(14) $$v_{i-1,m}^{\mathrm{npv}}(t+\Delta t)=v_{i-1,m}^{\mathrm{npv}}(t)+a_{i-1,m}^{\mathrm{npv}}(t)\Delta t$$

(15) $$x_{i-1,m}^{\mathrm{npv}}(t+\Delta t)=x_{i-1,m}^{\mathrm{npv}}(t)+v_{i-1,m}^{\mathrm{npv}}(t)\Delta t+\frac{1}{2}a_{i-1,m}^{\mathrm{npv}}(t)\Delta t^{2}$$

(4) State IV: the leading vehicle experiences queuing, while the following vehicle does not.

As shown in Figure 3, in Region IV, the leading vehicle $V_{i-1}^{\mathrm{pv}}$ has experienced queuing upon arrival, while the following vehicle $V_{i}^{\mathrm{pv}}$ has not. The trajectories requiring reconstruction can also be categorized into two types: those of vehicles that have not experienced queuing and those that have. The trajectories of vehicles that have not experienced queuing can be reconstructed using Equation (10), while the first-phase trajectories of vehicles that have experienced queuing can be reconstructed using the following equation [27]:

(16) $$\begin{array}{l} \min location\;error=\sqrt{{\left({\hat{x}}_{i}^{\mathrm{pv}}(t-2\Delta t)-x_{i}^{\mathrm{pv}}(t-2\Delta t)\right)}^{2}}\\ s.t.\;{\hat{a}}_{i}^{\mathrm{pv}}(t-\Delta t)=a_{\max}\left[1-{\left(\frac{v_{i}^{\mathrm{pv}}(t-\Delta t)}{v_{0}}\right)}^{4}-{\left(\frac{s^{*}(v_{i}^{\mathrm{pv}}(t-\Delta t),{\hat{v}}_{i-1,m}^{\mathrm{npv}}(t-\Delta t))}{{\hat{x}}_{i-1,m}^{\mathrm{npv}}(t-\Delta t)-x_{i}^{\mathrm{pv}}(t-\Delta t)}\right)}^{2}\right]\\ \max(v_{i-1,m}^{\mathrm{npv}}(t)-a_{\max},0)\leq {\hat{v}}_{i-1,m}^{\mathrm{npv}}(t-\Delta t)\leq \min(v_{i-1,m}^{\mathrm{npv}}(t)+a_{\max},v_{0})\\ {\hat{x}}_{i-1,m}^{\mathrm{npv}}(t-\Delta t)=x_{i-1,m}^{\mathrm{npv}}(t)+\frac{1}{2}({\hat{v}}_{i-1,m}^{\mathrm{npv}}(t-\Delta t)+v_{i-1,m}^{\mathrm{npv}}(t))\Delta t\\ {\hat{x}}_{i}^{\mathrm{pv}}(t-2\Delta t)=x_{i}^{\mathrm{pv}}(t-\Delta t)+v_{i}^{\mathrm{pv}}(t-\Delta t)\Delta t+\frac{1}{2}{\hat{a}}_{i}^{\mathrm{pv}}(t-\Delta t)\Delta t^{2} \end{array}$$

The second-phase trajectories of vehicles that have experienced queuing can be calculated using Equations (7)–(9). The third-phase trajectories can be reconstructed using the method for “State I”.

### 4.3. Vehicle Trajectory Self-Optimization Algorithm

The initial full-sample vehicle trajectories can be reconstructed using the four trajectory estimation algorithms. However, since the number of vehicles is estimated based on the minimum standstill spacing, the vehicle distribution tends to be uniform, which may introduce flow and positional errors under different trajectory states.

To effectively reduce errors, address data format incompatibilities, and improve the accuracy and smoothness of trajectory reconstruction, a particle filter algorithm is employed to optimize the initial full-sample vehicle trajectories. This optimization is further refined using upstream and downstream section data from fixed sensors for calibration. Due to its advantages in handling nonlinear and non-Gaussian systems, the particle filter algorithm has become a widely adopted approach in the fields of trajectory reconstruction and prediction, providing robust support for reconstructing dynamic behaviors in complex traffic flows [8,23].

(1) State–Space Model

The particle filter represents probability distributions using random sampling and updates the system state probabilities based on observed measurements. At time step *t*, $x_{i}(t)$ denotes the discrete state vector, and $z_{i}(t)$ represents the observation vector. The particle filter model is formulated as follows:

(17) $$x_{i}(t)=f(x_{i}(t-\Delta t),w_{i}(t-\Delta t))$$

(18) $$z_{i}(t)=h(x_{i}(t),s_{i}(t))$$

where f(⋅) represents the state transition function, h(⋅) represents the observation function, $w_{i}(t-\Delta t)$ denotes Gaussian system noise, and $s_{i}(t)$ denotes Gaussian observation noise.

In this study, the trajectory of each undetected vehicle in the reconstruction region is treated as a discrete state, with state variables defined by the position, velocity, and acceleration at time step *t*. The state vector is expressed as $x_{i}(t)={\left[x_{i}(t),v_{i}(t),a_{i}(t)\right]}^{⊤}$. The discrete state model is established as follows:

(19) $$x_{i}(t)=x_{i}(t-\Delta t)+\int_{t-\Delta t}^{t}f(x_{i}(\tau ))d\tau +w_{i}$$

The observation vector at time step *t* is:

(20) $$z_{i}(t)=\left[\begin{array}{c} Q_{\mathrm{up}}\\ v_{\mathrm{up}}(t)\\ Q_{\mathrm{down}}\\ v_{\mathrm{down}}(t) \end{array}\right]+s_{i}(t)$$

where $Q_{\mathrm{up}}$ and $Q_{\mathrm{down}}$ represent the traffic flow rates at the upstream and downstream sections, respectively, while $v_{\mathrm{up}}(t)$ and $v_{\mathrm{down}}(t)$ denote the detected speeds at the upstream and downstream sections, respectively.

(2) Optimizing Trajectories by Adjusting Particle Weights: The particle weights are calculated based on the predicted particle states and the observed values from upstream and downstream fixed sensors.

(21) $$w_{i}^{\left(p\right)}(t)\propto p(z_{i}(t)|x_{i}^{\left(p\right)}(t))$$

The observation probability is calculated based on a Gaussian distribution.

(22) $$p(z_{i}(t)|x_{i}^{\left(p\right)}(t))=\frac{1}{\sqrt{{\left(2\pi \right)}^{d}|\Sigma_{v}|}}\exp\left\lfloor -\frac{1}{2}{\left(z_{i}(t)-h(x_{i}^{\left(p\right)}(t))\right)}^{⊤}\Sigma_{v}^{-1}(z_{i}(t)-h(x_{i}^{\left(p\right)}(t)))\right\rfloor$$

Based on the particle weight distribution ${\left\{w_{i}^{\left(p\right)}(t)\right\}}_{p=1}^{N_{p}}$, resampling is performed, and the optimal trajectory is estimated using the weighted average of the particles. The optimization objective is defined as minimizing the error between the reconstructed trajectory positions and the actual positions, allowing for iterative trajectory refinement.

## 5. Case Study

### 5.1. Scenario Description and Performance Indicators

**Figure 5:**
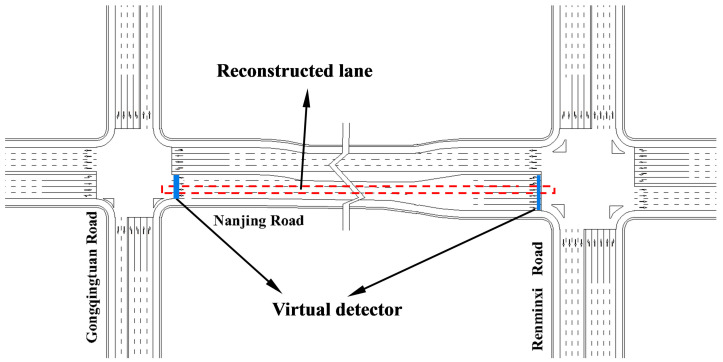
Layout of the studied road segment.

To validate the effectiveness of the proposed method under different traffic conditions, this study selected Nanjing Road in Zibo City, specifically the section from Gongqingtuan West Road to Renmin West Road, as the study area. A simulation road network model was established using the simulation software TESSNG [34]. The studied road segment is 530 m long, features six lanes in both directions, and includes a downstream intersection controlled by a four-phase fixed-time traffic signal. The downstream segment consists of two left-turn lanes, three through lanes, and one right-turn lane. For trajectory reconstruction, one through lane on Nanjing Road was selected as the reconstruction region, as shown in Figure 5.

Based on the detection data from the upstream and downstream electric monitoring points and the downstream signal timing data collected on 3 January 2024, the relevant parameters of the model were calibrated. On this basis, vehicle trajectory data within the road segment were simulated and collected using the TESSNG simulation software to serve as the validation dataset for the model. The simulation was configured with a duration of 1 h (approximately 23 signal cycles), with the first two signal cycles designated as the warm-up phase. Additionally, the trajectory dataset was analyzed, calculated, and processed to extract the model parameters, as shown in Table 1.

Figure 5 illustrates the study area and the locations of the upstream and downstream virtual fixed sensors. Since the reconstruction results consist of individual vehicle trajectories, certain macroscopic metrics, such as travel time and delay, are not applicable. Therefore, this study adopts three microscopic metrics—Mean Absolute Error (MAE), Mean Absolute Percentage Error (MAPE), and root-mean-square error (RMSE)—to comprehensively evaluate the proposed method. These three metrics have been widely used to assess reconstruction accuracy [15,31], with their calculations based on a second-by-second comparison between the actual and reconstructed trajectories.

(23) $$\mathrm{MAE}=\frac{1}{N}\sum_{i=1}^{N}\left|x_{i}^{actual}-x_{i}^{reconstructed}\right|$$

(24) $$\mathrm{MAPE}=\frac{1}{N}\sum_{i=1}^{N}\left|\frac{x_{i}^{actual}-x_{i}^{reconstructed}}{x_{i}^{actual}}\right|*100\%$$

(25) $$\mathrm{RMSE}=\sqrt{\frac{1}{N}{\sum_{i=1}^{N}\left(x_{i}^{actual}-x_{i}^{reconstructed}\right)}^{2}}$$

where $x_{i}^{actual}$ and $x_{i}^{reconstructed}$ represent the actual trajectory position and the reconstructed trajectory position at time *i*, respectively. MAE denotes the Mean Absolute Error, which represents the average absolute positional error; MAPE refers to the Mean Absolute Percentage Error, which quantifies the percentage error of the reconstructed trajectory relative to the actual value; and RMSE represents the root-mean-square error, which measures the root mean square of the differences between actual and reconstructed trajectory positions and is particularly sensitive to outliers.

### 5.2. Results and Discussion

#### 5.2.1. General Results Analysis

**Figure 6:**
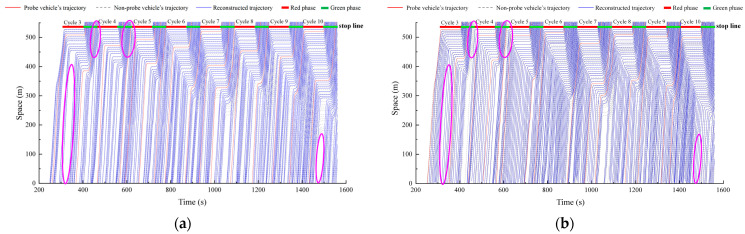
Reconstructed trajectories of the proposed method and the PV method: (**a**) the reconstructed trajectories of the proposed method; (**b**) the reconstructed trajectories of the PV method.

To verify the accuracy of the proposed method in vehicle trajectory reconstruction, a comparative analysis was conducted with a trajectory reconstruction method based on PVs (the PV method). The PV method uses the trajectory data of PVs and signal timing data as inputs and reconstructs trajectories solely based on the trajectory state estimation algorithm without incorporating a trajectory self-optimization process. In contrast, the proposed method takes as inputs the trajectory data of PVs, signal timing data, and upstream and downstream fixed sensor data. In Figure 6, the trajectories of PVs are represented by red lines, the actual trajectories of non-probe vehicles are shown as gray dashed lines, and the reconstructed trajectories of non-probe vehicles are depicted with blue lines. Due to space limitations, only the spatiotemporal trajectories of eight signal cycles are presented.

From the reconstructed vehicle trajectories, the proposed method demonstrates a relatively high level of accuracy in reconstructing the trajectories of non-probe vehicles for each signal cycle. The PV method also performs well in each cycle. However, the PV method overlooks the randomness of vehicle arrivals and departures on urban road segments and, due to the low penetration rate of PVs, fails to accurately determine the number of non-probe vehicles, as indicated by the pink circles in Figure 6a,b.

To comprehensively compare the performance of the two methods in terms of trajectory reconstruction accuracy, a detailed comparative analysis of three error metrics—MAE, MAPE, and RMSE—was conducted, as shown in Table 2. The results demonstrate that the proposed method achieved MAE, MAPE, and RMSE values of 12.64 m, 4.35%, and 14.56 m, respectively, representing reductions of 14.31%, 22.87%, and 13.36% compared to the PV method. This indicates that the proposed trajectory reconstruction method based on multi-source data fusion significantly outperforms the PV method in terms of reconstruction accuracy, further confirming the effectiveness and applicability of multi-source data fusion in improving trajectory reconstruction accuracy.

This improvement is consistent with the findings of existing studies in the field of multi-source data fusion, further confirming the potential of data fusion in enhancing trajectory reconstruction accuracy. For instance, Zha et al. (2025) [17,35] proposed a deep learning-based multimodal data fusion method that effectively integrates synergistic information among multiple sensors and establishes mapping relationships between different data sources, thereby improving the environmental perception capability of autonomous vehicles. However, the implementation of this method relies heavily on substantial computational resources and high-quality data, which limits its applicability in sparse traffic data environments. In contrast, the proposed method maintains high reconstruction accuracy even under lower probe vehicle penetration rates and more complex traffic conditions, demonstrating strong robustness and adaptability. High-precision full-sample vehicle trajectory data can provide valuable real-time support for traffic signal control optimization, traffic anomaly detection and warning, and traffic flow analysis.

Moreover, Deng et al. (2023) [36] proposed a method that integrates probe vehicle data and fixed sensor data, which has improved the accuracy of vehicle trajectory estimation in multi-lane traffic scenarios to a certain extent. However, due to the failure to fully consider the issue of data sparsity caused by the low penetration rate of probe vehicles, there remains a certain degree of deviation in trajectory estimation, and the reconstruction accuracy is still limited. In contrast, the proposed method effectively leverages the advantages of fixed sensors through a trajectory self-optimization algorithm, which compensates for the trajectory estimation errors caused by the low penetration rate of probe vehicles. As a result, the proposed method demonstrates superior adaptability in complex traffic environments.

Banani Ardecani et al. (2024) [37] further proposed a multi-sensor data fusion method that effectively integrates different types of sensor data, significantly improving the accuracy of travel time estimation and enhancing the overall perception of traffic flow conditions. However, this study primarily focused on the perception and estimation of macroscopic traffic states, with a limited consideration of the detailed reconstruction of vehicle trajectories at the microscopic level. Consequently, there may be limitations in reconstruction accuracy under complex traffic scenarios. In contrast, the proposed method, by combining a trajectory self-optimization algorithm with multi-source data fusion, not only performs well in macroscopic traffic state estimation but also achieves higher accuracy in microscopic trajectory reconstruction, further demonstrating the effectiveness and applicability of the proposed method.

#### 5.2.2. Analysis of the Impact of Traffic Density

**Figure 7:**
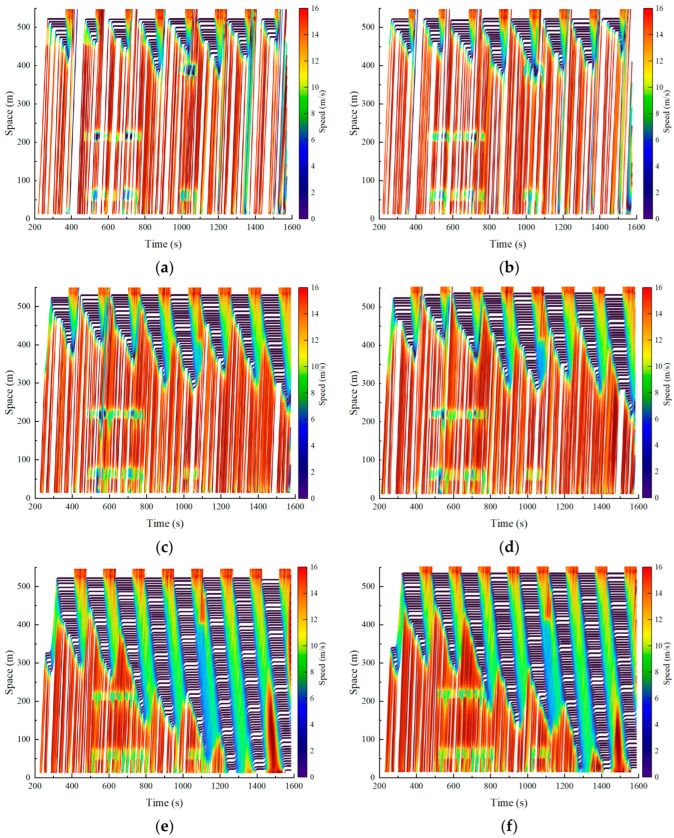
Reconstructed trajectories under different traffic density conditions: (**a**) actual trajectories under low-density conditions; (**b**) reconstructed trajectories under low-density conditions; (**c**) actual trajectories under medium-density conditions; (**d**) reconstructed trajectories under medium-density conditions; (**e**) actual trajectories under high-density conditions; (**f**) reconstructed trajectories under high-density conditions.

The traffic density on urban roads directly affects the distribution characteristics of vehicles and the complexity of trajectory reconstruction. To evaluate the performance of the proposed method under different traffic densities, the penetration rate of PV was set to 10%. The reconstruction accuracy of the proposed method was tested under three typical traffic density conditions, low density (30 veh/km), medium density (40 veh/km), and high density (50 veh/km) [15], as illustrated in Figure 7.

To better evaluate the effectiveness of the proposed method for trajectory reconstruction in a multi-source data environment, a comparison was conducted between the trajectory reconstruction method based on PVs (the PV method) and the trajectory reconstruction method based on fixed sensors (the FS method) [22]. The PV method relies entirely on the car-following model to reconstruct vehicle trajectories. In contrast, the FS method generates undetected vehicle trajectories by integrating fixed detector data and traffic signal timing information, leveraging traffic wave theory and traffic simulation techniques.

As shown in Figure 7, the proposed method effectively reconstructs vehicle travel trajectories under varying traffic densities and accurately captures the dynamic characteristics of vehicle movements. Additionally, the more refined trajectories provide robust support for an in-depth analysis of microscopic traffic flow patterns [15]. However, traffic density significantly influences reconstruction accuracy. As presented in Table 3, when traffic density increases from 30 veh/km to 50 veh/km, the MAE decreases from 15.49 m to 9.41 m, the MAPE decreases from 5.78% to 3.63%, and the RMSE decreases from 18.37 m to 12.49 m. This improvement can be attributed to the proposed method’s reliance on the car-following model. As traffic density increases, the reduced spacing between vehicles enhances mutual interactions, resulting in more pronounced car-following behavior. Consequently, the proposed method demonstrates effective trajectory reconstruction across different traffic densities, with reconstruction accuracy progressively improving as traffic density increases.

According to the data in Table 3, the trajectory reconstruction errors of all three methods exhibit an overall decreasing trend as traffic density increases. Among these, the proposed method consistently achieves the best performance across different traffic density conditions. In contrast, the FS method performs poorly under medium- and high-density conditions; however, its errors significantly decrease with increasing traffic density. Under low-density conditions, the FS method outperforms the PV method, with MAE, MAPE, and RMSE values of 21.15 m, 7.95%, and 25.82 m, respectively, compared to 23.35 m, 8.89%, and 28.24 m for the PV method. This advantage can be attributed to the ability of fixed sensors to capture comprehensive information on all passing vehicles under low-density conditions. In contrast, the PV method is constrained by speed fluctuations and limited data coverage, resulting in incomplete data collection and higher reconstruction errors.

#### 5.2.3. An Analysis of the Impact of the PV Penetration Rate

**Figure 8:**
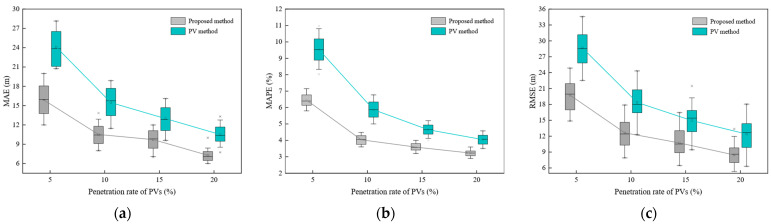
Boxplots of two methods under different PV penetration rates: (**a**) MAE under different penetration rates; (**b**) MAPE under different penetration rates; (**c**) RMSE under different penetration rates.

The PV penetration rate is a critical factor influencing the accuracy of trajectory reconstruction, as its variation directly determines the data coverage available to the model and the complexity of trajectory completion. To evaluate the performance of the proposed method under different PV penetration rates, the traffic flow density was set to 40 veh/km. The reconstruction accuracy of the proposed method and the PV method was examined under four PV penetration rates: 5%, 10%, 15%, and 20%. The error boxplots for both methods are presented in Figure 8, and the detailed trajectory reconstruction results are shown in Table 4.

As shown in Figure 8, the proposed method consistently achieves significantly higher trajectory reconstruction accuracy than the PV method across all penetration rate conditions, while also demonstrating greater stability. As the PV penetration rate increases from 5% to 20%, the error metrics (MAE, MAPE, and RMSE) for both methods show a significant decreasing trend. However, compared to the PV method, the proposed method exhibits substantially smaller error fluctuations, indicating more stable performance. Notably, under low penetration rate conditions, the proposed method demonstrates a particularly pronounced performance advantage, highlighting its ability to effectively reconstruct the trajectories of non-probe vehicles in sparse data environments, thereby showcasing its superior adaptability.

As shown in Table 4, when the penetration rate is 5%, the proposed method achieves an MAE of 16.25 m, an MAPE of 6.52%, and an RMSE of 19.87 m, representing reductions of 33.78%, 34.07%, and 32.30%, respectively, compared to the PV method, which has values of 24.54 m, 9.89%, and 29.35 m. At a penetration rate of 20%, the proposed method’s MAE, MAPE, and RMSE are 7.35 m, 3.13%, and 8.36 m, respectively, showing reductions of 15.22%, 18.91%, and 17.72% compared to the PV method. These results indicate that the penetration rate significantly influences the performance of both the PV method and the proposed method. This analysis revealed that the PV method exhibits a strong dependency on the PV penetration rate [31,33], resulting in larger errors under low penetration conditions, which limits its ability to effectively capture the dynamic behaviors of non-probe vehicles. In contrast, the proposed method demonstrates smaller error fluctuations and higher stability at higher penetration rates, making it more suitable for trajectory reconstruction in complex traffic scenarios.

**Figure 9:**
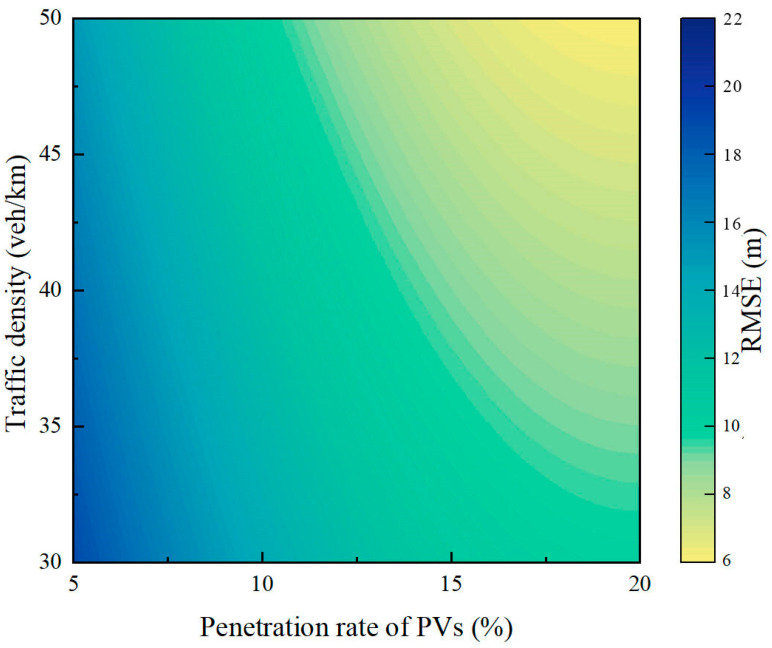
Heatmap of root-mean-square error.

Additionally, this study further analyzed the impact of traffic density and the PV penetration rate on the performance of the proposed model, and the corresponding RMSE heatmap is presented in Figure 9. In this heatmap, the *X*-axis represents the effect of the PV penetration rate on model accuracy, while the *Y*-axis represents the effect of traffic density on model accuracy.

As shown in Figure 9, the RMSE of the model exhibits a decreasing trend as the PV penetration rate or traffic density increases, leading to improved trajectory reconstruction accuracy and performance. Comparatively, the PV penetration rate has a more significant impact on model accuracy. The analysis indicated that an increase in the PV penetration rate allows more vehicles to provide high-resolution trajectory data, such as position, velocity, and acceleration, which aligns with findings from existing studies [15,33]. These high-resolution data can be directly used in the model to complement the trajectories of non-probe vehicles, exerting a more fundamental and comprehensive influence on trajectory reconstruction quality. In contrast, traffic density primarily affects trajectory reconstruction indirectly by influencing vehicle behavior patterns, resulting in a relatively smaller impact. Therefore, higher PV penetration rates lead to more precise model inputs and lower reconstruction errors.

## 6. Conclusions

This study proposes a full-sample vehicle trajectory reconstruction method for urban arterials by integrating fixed sensor and probe vehicle (PV) data, overcoming limitations in spatial coverage and trajectory continuity under low sensor deployment and PV penetration rates. The main conclusions are as follows:

1. This study designed four trajectory state estimation algorithms based on the driving states of neighboring PVs, effectively identifying the spatiotemporal interactions between probe and non-probe vehicles and achieving complete reconstruction of non-probe vehicle trajectories.
2. A trajectory self-optimization algorithm based on particle filtering was proposed to minimize position errors. By integrating data from upstream and downstream fixed sensors with initial full-sample trajectories, the algorithm resolves data format incompatibility and randomness issues, improving the smoothness and reliability of trajectory reconstruction.
3. Case studies showed that the proposed method improves trajectory accuracy by an average of 16.85% over the PV method. The MAPE is reduced by 2.28% under high-density conditions and by 3.37% under low penetration rates, demonstrating the method’s superior robustness and adaptability in complex traffic environments.
4. A comprehensive evaluation revealed that reconstruction accuracy improves consistently with increasing traffic density and PV penetration rates. PV penetration has a greater impact on model accuracy than traffic density.

This method enhances the accuracy of full-sample vehicle trajectory reconstruction on urban arterials, providing valuable insights for traffic flow analysis and intelligent transportation system optimization.

However, the model still has the following limitations:

1. This study mainly focused on the longitudinal interaction behavior of vehicles and did not fully consider the impact of lateral vehicle interactions in multi-lane environments.
2. This study mainly relied on data from fixed sensors and probe vehicles, without fully utilizing the advantages of other potential data sources (such as drone monitoring and high-precision maps).

In future research, we will integrate multi-lane lane-changing behavior and multimodal data to further improve the adaptability and reconstruction accuracy of the model, thereby advancing the development and application of intelligent transportation systems.

## Figures and Tables

**Table 1:** Model parameters.

Variable	Description	Value
$v_{0}$	Desired velocity (m/s)	16
T	Safe time headway (s)	1.5
$a_{\max}$	Maximum acceleration (m/s^2^)	5
b	Comfortable deceleration (m/s^2^)	2
$s_{0}$	Minimum distance (m)	2

**Table 2:** Comparison of results between the proposed method and the PV method.

No. of Cycle	Reconstruction Method	MAE (m)	MAPE (%)	RMSE (m)
21	Proposed method	12.64	4.35	14.56
	PV method	14.75	5.64	17.96
	Comparison	−14.31%	−22.87%	−13.36%

**Table 3:** Trajectory reconstruction results of three methods under different traffic density conditions.

Traffic Density (veh/km)	Reconstruction Method	MAE (m)	MAPE (%)	RMSE (m)
30	Proposed method	15.49	5.78	18.37
	PV method	23.35	8.89	28.24
	FS method	21.15	7.95	25.82
40	Proposed method	10.73	4.05	12.89
	PV method	14.62	5.65	17.45
	FS method	16.51	6.24	20.72
50	Proposed method	9.41	3.63	12.49
	PV method	12.75	4.63	14.31
	FS method	15.66	5.91	18.72

**Table 4:** Trajectory reconstruction results of two methods under different PV penetration rates.

Penetration Rate of PVs (%)	Reconstruction Method	MAE (m)	MAPE (%)	RMSE (m)
5	Proposed method	16.25	6.52	19.87
	PV method	24.54	9.89	29.35
	Comparison	−33.78%	−34.07	−32.30%
10	Proposed method	10.73	4.05	12.89
	PV method	14.62	5.65	17.45
	Comparison	−26.61%	−28.32%	−26.13%
15	Proposed method	9.86	3.52	11.45
	PV method	11.78	4.36	14.13
	Comparison	−16.30%	−19.27%	−18.97%
20	Proposed method	7.35	3.13	8.36
	PV method	8.67	3.86	10.16
	Comparison	−15.22%	−18.91%	−17.72%

## Data Availability

Some of the data in this study are contained within the article, and additional data are available upon request from the corresponding author.

## References

[1] Wang J., Fu T., Shangguan Q. (2023). Wide-Area Vehicle Trajectory Data Based on Advanced Tracking and Trajectory Splicing Technologies: Potentials in Transportation Research. Accid. Anal. Prev..

[2] Guo Q., Li L., Ban X.J. (2019). Urban Traffic Signal Control with Connected and Automated Vehicles: A Survey. Transp. Res. Part C Emerg. Technol..

[3] Li L., Jiang R., He Z., Chen X.M., Zhou X. (2020). Trajectory Data-Based Traffic Flow Studies: A Revisit. Transp. Res. Part C Emerg. Technol..

[4] Barmpounakis E., Geroliminis N. (2020). On the New Era of Urban Traffic Monitoring with Massive Drone Data: The *pNEUMA* Large-Scale Field Experiment. Transp. Res. Part C Emerg. Technol..

[5] Mehran B., Kuwahara M., Naznin F. (2012). Implementing Kinematic Wave Theory to Reconstruct Vehicle Trajectories from Fixed and Probe Sensor Data. Transp. Res. Part C Emerg. Technol..

[6] Zheng J., Liu H.X. (2017). Estimating Traffic Volumes for Signalized Intersections Using Connected Vehicle Data. Transp. Res. Part C Emerg. Technol..

[7] Tan C., Liu L., Wu H., Cao Y., Tang K. (2020). Fuzing License Plate Recognition Data and Vehicle Trajectory Data for Lane-Based Queue Length Estimation at Signalized Intersections. J. Intell. Transp. Syst..

[8] Wei L., Wang Y., Chen P. (2021). A Particle Filter-Based Approach for Vehicle Trajectory Reconstruction Using Sparse Probe Data. IEEE Trans. Intell. Transp. Syst..

[9] Gong J., Peng X. (2014). Determining Traffic State Evolution Index on Urban Arterial Road. J. Transp. Syst. Eng. Inf. Technol..

[10] Wang Y., Papageorgiou M., Messmer A. (2008). Real-Time Freeway Traffic State Estimation Based on Extended Kalman Filter: Adaptive Capabilities and Real Data Testing. Transp. Res. Part A Policy Pract..

[11] Yao J., Li F., Tang K., Jian S. (2020). Sampled Trajectory Data-Driven Method of Cycle-Based Volume Estimation for Signalized Intersections by Hybridizing Shockwave Theory and Probability Distribution. IEEE Trans. Intell. Transp. Syst..

[12] Seo T., Kawasaki Y., Kusakabe T., Asakura Y. (2019). Fundamental Diagram Estimation by Using Trajectories of Probe Vehicles. Transp. Res. Part B Methodol..

[13] Yin J., Chen P., Tang K., Sun J. (2021). Queue Intensity Adaptive Signal Control for Isolated Intersection Based on Vehicle Trajectory Data. J. Adv. Transp..

[14] Sun Z., Hao P., Ban X., Yang D. (2015). Trajectory-Based Vehicle Energy/Emissions Estimation for Signalized Arterials Using Mobile Sensing Data. Transp. Res. Part D Transp. Environ..

[15] Chen X., Yin J., Qin G., Tang K., Wang Y., Sun J. (2022). Integrated Macro-Micro Modelling for Individual Vehicle Trajectory Reconstruction Using Fixed and Mobile Sensor Data. Transp. Res. Part C Emerg. Technol..

[16] Chen P., Wei L., Meng F., Zheng N. (2021). Vehicle Trajectory Reconstruction for Signalized Intersections: A Hybrid Approach Integrating Kalman Filtering and Variational Theory. Transp. B Transp. Dyn..

[17] Eskandarian A., Wu C., Sun C. (2021). Research Advances and Challenges of Autonomous and Connected Ground Vehicles. IEEE Trans. Intell. Transp. Syst..

[18] Tang K., Tan C., Cao Y., Yao J., Sun J. (2020). A Tensor Decomposition Method for Cycle-Based Traffic Volume Estimation Using Sampled Vehicle Trajectories. Transp. Res. Part C Emerg. Technol..

[19] Zhang H., Liu H.X., Chen P., Yu G., Wang Y. (2020). Cycle-Based End of Queue Estimation at Signalized Intersections Using Low-Penetration-Rate Vehicle Trajectories. IEEE Trans. Intell. Transp. Syst..

[20] Chen P., Zeng W., Chen M., Yu G., Wang Y. (2019). Modeling Arterial Travel Time Distribution by Accounting for Link Correlations: A Copula-Based Approach. J. Intell. Transp. Syst..

[21] Rao W., Wu Y.-J., Xia J., Ou J., Kluger R. (2018). Origin-Destination Pattern Estimation Based on Trajectory Reconstruction Using Automatic License Plate Recognition Data. Transp. Res. Part C Emerg. Technol..

[22] van Lint J.W.C., Hoogendoorn S.P. (2010). A Robust and Efficient Method for Fusing Heterogeneous Data from Traffic Sensors on Freeways. Comput.-Aided Civ. Infrastruct. Eng..

[23] Xie X., van Lint H., Verbraeck A. (2018). A Generic Data Assimilation Framework for Vehicle Trajectory Reconstruction on Signalized Urban Arterials Using Particle Filters. Transp. Res. Part C Emerg. Technol..

[24] Goodall N.J., Smith B.L., Park B.B. (2016). Microscopic Estimation of Freeway Vehicle Positions from the Behavior of Connected Vehicles. J. Intell. Transp. Syst..

[25] Wang Y., Wei L., Chen P. (2020). Trajectory Reconstruction for Freeway Traffic Mixed with Human-Driven Vehicles and Connected and Automated Vehicles. Transp. Res. Part C Emerg. Technol..

[26] Chen P., Wang T., Zheng N. (2022). Reconstructing Vehicle Trajectories on Freeways Based on Motion Detection Data of Connected and Automated Vehicles. J. Intell. Transp. Syst..

[27] Chen X., Yin J., Tang K., Tian Y., Sun J. (2022). Vehicle Trajectory Reconstruction at Signalized Intersections Under Connected and Automated Vehicle Environment. IEEE Trans. Intell. Transp. Syst..

[28] Ounoughi C., Ben Yahia S. (2023). Data Fusion for ITS: A Systematic Literature Review. Inf. Fusion.

[29] Liu X., Zhang Z., Miwa T., Cao P. (2023). Estimating Freeway Lane-Level Traffic State with Intelligent Connected Vehicles. Transp. Res. Rec..

[30] Zhang J., Huang D., Liu Z., Zheng Y., Han Y., Liu P., Huang W. (2024). A Data-Driven Optimization-Based Approach for Freeway Traffic State Estimation Based on Heterogeneous Sensor Data Fusion. Transp. Res. Part E Logist. Transp. Rev..

[31] Chen X., Qin G., Seo T., Yin J., Tian Y., Sun J. (2024). A Macro-Micro Approach to Reconstructing Vehicle Trajectories on Multi-Lane Freeways with Lane Changing. Transp. Res. Part C Emerg. Technol..

[32] Ji H., Mei J., Wang L., Liu S., Ren Y. (2023). Data-Driven Kalman Consensus Filtering for Connected Vehicle Speed Estimation in a Multi-Sensor Network. Symmetry.

[33] Yao Z., Liu M., Jiang Y., Tang Y., Ran B. (2024). Trajectory Reconstruction for Mixed Traffic Flow with Regular, Connected, and Connected Automated Vehicles on Freeway. IET Intell. Transp. Syst..

[34] Shi Y., Wang D., Liu B., Deng M., Chen B. (2024). Exploring the Nonlinear Relationships between Human Travel and Road Traffic Congestions Using Taxi Trajectory Data. Transportation.

[35] Zha L., Gong C., Lv K. (2025). Real-Time Localization and Navigation Method for Autonomous Vehicles Based on Multi-Modal Data Fusion by Integrating Memory Transformer and DDQN. Image Vis. Comput..

[36] Deng Y., Cao Q., Ren G., Ma J., Zhu S. (2023). Vehicle Trajectory Reconstruction Incorporating Probe and Fixed Sensor Data. J. Transp. Eng. Part A Syst..

[37] Banani Ardecani F., Mahmoudzadeh A., Mesbah M. (2024). Fuzing Multiple Erroneous Sensors to Estimate Travel Time. J. Intell. Transp. Syst..

